# Base modifications affecting RNA polymerase and reverse transcriptase fidelity

**DOI:** 10.1093/nar/gky341

**Published:** 2018-05-10

**Authors:** Vladimir Potapov, Xiaoqing Fu, Nan Dai, Ivan R Corrêa, Nathan A Tanner, Jennifer L Ong

**Affiliations:** 1 New England Biolabs, Inc, Ipswich, Massachusetts, 01938, USA; 2 Dalian University of Technology, School of Life Science and Biotechnology, Dalian, Liaoning 116021, China

## Abstract

Ribonucleic acid (RNA) is capable of hosting a variety of chemically diverse modifications, in both naturally-occurring post-transcriptional modifications and artificial chemical modifications used to expand the functionality of RNA. However, few studies have addressed how base modifications affect RNA polymerase and reverse transcriptase activity and fidelity. Here, we describe the fidelity of RNA synthesis and reverse transcription of modified ribonucleotides using an assay based on Pacific Biosciences Single Molecule Real-Time sequencing. Several modified bases, including methylated (m^6^A, m^5^C and m^5^U), hydroxymethylated (hm^5^U) and isomeric bases (pseudouridine), were examined. By comparing each modified base to the equivalent unmodified RNA base, we can determine how the modification affected cumulative RNA polymerase and reverse transcriptase fidelity. 5-hydroxymethyluridine and *N^6^*-methyladenosine both increased the combined error rate of T7 RNA polymerase and reverse transcriptases, while pseudouridine specifically increased the error rate of RNA synthesis by T7 RNA polymerase. In addition, we examined the frequency, mutational spectrum and sequence context of reverse transcription errors on DNA templates from an analysis of second strand DNA synthesis.

## INTRODUCTION

In addition to the canonical nucleobases, RNA molecules are capable of hosting a variety of chemically diverse modifications. Over 100 naturally occurring post-transcriptional modifications have been identified so far (reviewed and discussed in (1,2) and catalogued in The RNA Modification Database (3)). The most extensive chemical diversity is seen in transfer RNA, while ribosomal RNA, noncoding RNA and viral RNA genomes also contain a substantial number of modifications. Messenger RNA from a variety of organisms, including eukaryotes, contains internal modifications such as *N^6^*-methyladenosine (m^6^A), 5-methylcytidine (m^5^C), pseudouridine (Ψ), 5-hydroxymethylcytidine (hm^5^C) and inosine (I) (4). Modifications can alter gene expression or mRNA stability, and were found to be conserved, regulated, and implicated in various cellular, developmental and disease processes (5). Modified RNA bases also reduce the immunogenicity of therapeutic RNA (6). Next-generation sequencing technologies have advanced the study of RNA modifications and enabled transcriptome-wide mapping of modified bases at single-base resolution (7–13). However, in spite of the vast chemical diversity, biological significance and therapeutic potential, little is known about the accuracy of incorporation or reverse transcription of modified RNA bases.

Various methods have been used to study the accuracy of RNA polymerases and reverse transcriptases, including cell-based phenotypic assays, enzyme kinetics, Sanger sequencing, and next-generation sequencing (14–23). Next-generation sequencing, especially methods and technologies that enable accurate sequencing of individual molecules, is especially useful for measuring rare replication errors in large sequencing data sets. However, measuring replication errors in RNA presents its own challenges, requiring conversion of RNA to DNA by reverse transcriptases, as current high-accuracy next-generation sequencing technologies only sequence DNA templates. Thus, for most fidelity assays, the measured error rate is typically the combined error rate of both the RNA polymerase and reverse transcriptase used to create the DNA library to be interrogated. In an elegant solution, Gout and colleagues were able to address this problem by tagging individual RNA transcripts with molecular indexes and reverse transcribing multiple copies of each transcript prior to Illumina sequencing, thus separately detecting *in vivo* transcription errors and errors arising from reverse transcription or library preparation (24). A similar barcoding strategy using rolling-circle reverse transcription, was recently used to study transcriptional mutagenesis in yeast (25,26). Although multiple studies have examined transcription errors and reverse transcriptase fidelity, only a few have examined the effect of base modification (27–29), and consequently, how RNA modifications affect RNA sequencing data.

In the present study, we developed an assay based on Pacific Biosciences Single Molecule Real-Time (SMRT) sequencing to measure the fidelity of transcription and reverse transcription of modified and unmodified RNA. T7 RNA polymerase synthesized RNA from nucleotide pools containing *N*^6^-methyladenosine (m^6^A), pseudouridine (Ψ), 5-methylcytidine (m^5^C), 5-methyluridine (m^5^U, or ribonucleoside thymidine) and 5-hydroxymethyluridine (hm^5^U) triphosphates. After synthesis of modified RNA, first and second strand reverse transcriptase error rates were measured by SMRT sequencing. SMRT sequencing can be used to generate very accurate sequencing data with a low background rate of substitution errors (30). True replication errors can be distinguished from sequencing errors by circular consensus sequencing, in which each individual circular template molecule is read multiple times. By comparing the cDNA strand to the reference sequence, we identified errors cumulatively made by T7 RNA polymerase and reverse transcriptase. Furthermore, because both strands are sequenced together, we were able to uniquely isolate mistakes made by the reverse transcriptase during synthesis of the second strand. We also compared the error rates of viral reverse transcriptases and *Bst* DNA polymerase variants with reverse transcriptase activity. In addition to analyzing the frequency and type of errors introduced during first and second strand synthesis, we also analyzed the sequence context around substitution events and how base modifications affected the turnover rate of reverse transcriptases.

## MATERIALS AND METHODS

All reagents are from New England Biolabs, unless otherwise stated. All oligonucleotides were synthesized by Integrated DNA Technologies.

### *In-vitro* transcription

Artificial sequences DNA-1 and DNA-2 (described in (30)), and DNA-3 and DNA-4 (detailed in the Supplementary Data), were cloned into a T7 vector, and linearized with HpaI (20 μg plasmid, 100 U HpaI, 1× ThermoPol Buffer in 1 ml total volume for 1 h at 37°C), then treated with PreCR (additional 20 μl PreCR Repair Mix, and a final concentration of 0.1 mM each dNTP, 0.5 mM NAD^+^ and 1× ThermoPol Buffer were added to the HpaI digest for total volume of 1.1 ml, and incubated for 30 min at 37°C). Transcription templates were cleaned using a Zymo DNA Clean & Concentrator-25 kit (Zymo Research). 5 μg linearized and repaired plasmids were transcribed using the HiScribe T7 High Yield RNA Synthesis kit (#E2040) and the Standard RNA Synthesis protocol in a total volume of 100 μl for 2 h at 37°C. For unmodified RNA, 10 mM each ATP, UTP, GTP and CTP were used. For modified RNA synthesis, the equivalent unmodified nucleoside triphosphate was replaced with 10 mM *N*^6^-methyladenosine-5′-triphosphate (#N-1013, all nucleoside triphosphates purchased from TriLink Biotechnologies), pseudouridine-5′-triphosphate (#N-1019), 5-methylcytidine-5′-triphosphate (#N-1014), 5-methyluridine-5′-triphosphate (#N-1024) or 5-hydroxymethyluridine-5′-triphosphate (#N-1086). After transcription, 4 U DNase I was added and incubated for 30 min at 37°C and RNA was purified using a MEGAclear Transcription Clean-Up Kit (ThermoFisher Scientific). RNA quantity and quality were assessed by running transcription products on a Bioanalyzer using a RNA 6000 Nano kit (Agilent Genomics) and a Novex 6% TBE–urea gel stained with 1× SYBR Gold Nucleic Acid Gel Stain (both from ThermoFisher Scientific). Incorporation efficiency of modified nucleoside triphosphates were determined by LC–MS or LC–MS/MS and found to be: 102% (m^6^A), 99% (Ψ), 95% (m^5^C), 104% (m^5^U) and 92% (hm^5^U) relative to guanosine (Supplementary Figure S1 and Supplementary Table S2).

### cDNA (first strand) synthesis and second strand synthesis

cDNA synthesis and the second strand synthesis were performed using the ProtoScript II First Strand cDNA Synthesis Kit (#E6560) with 10 μg RNA, 0.5 μM reverse primer, 1× ProtoScript II Reaction Mix and 1× ProtoScript II Enzyme Mix in 100 μl total reaction volume. Reactions were incubated for 1 h at 42°C. After cDNA synthesis, 20 U RNase H was added to the reaction and incubated for 1 h at 37°C. Single-stranded cDNA was purified using a Zymo DNA Clean & Concentrator-25 kit (Zymo Research) according to the manufacturer's protocol for purifying single-stranded DNA and with an elution volume of 44 μl. Second strand synthesis reactions (100 μl) contained: 40 μl purified cDNA, 5 μM forward primer, 1× ProtoScript II Reaction Mix and 1× ProtoScript II Enzyme Mix and incubated at 42°C for 1 h. Alternatively, cDNA and second strand synthesis were performed with M-MuLV Reverse Transcriptase (2.5 μg or 50 U) and 1× M-MuLV Reverse Transcriptase Reaction Buffer, or AMV Reverse Transcriptase (50 U) and 1× AMV Reverse Transcriptase Buffer. Synthesis products were purified with another Zymo column.

### PacBio SMRTbell library preparation and sequencing

Double-stranded DNA products were digested with 30 U BssSαI and 20 U DpnI in 1× CutSmart Buffer (100 μl total volume) for 1 h at 37°C and cleaned using a Zymo column. Digest products were ligated to 300 pmol SMRTbell adaptors with compatible BssSαI overhangs (/5Phos/TCGTATCTCTCTCTTTTCCTCCTCCTCCGTTGTTGTTGTTGAGAGAGAT-3′) with 800 U T4 DNA Ligase in 1× T4 DNA Ligase Reaction Buffer (50 μl total reaction volume) at room temperature for 30 min, followed by heat inactivation for 10 min at 65°C. Then, 50 U Exonuclease III (*Escherichia coli*) and 5 U Exonuclease VII were added to the reaction and incubated at 37°C for 1 h. SMRTbell libraries were cleaned using a Zymo column, then additionally size selected with AMPure PB beads (Pacific Biosciences) using 1× volume (500 bp libraries) or 0.6× volume (1 kb libraries). Pacific Biosciences Binding Calculator was used to generate a protocol for annealing sequencing primers and polymerase binding using the DNA/Polymerase Binding Kit P6 v2 (Pacific Biosciences) and default settings. DNA-1 and DNA-2 samples sequenced using the MagBead OCPW protocol (with 6 h movies) and DNA-3 and DNA-4 samples using the Standard protocol (diffusion loading with 5 h movies).

### Computational methods for determining error rates

High-accuracy consensus sequences were determined for the first and second strand for each sequenced double-stranded DNA as described in Potapov and Ong (30). The resulting high accuracy consensus sequences were mapped to the reference sequence using the BWA-MEM algorithm (31), and base substitutions, deletions, and insertions were determined along with the corresponding quality values. The following filtering steps were used to ensure the quality of consensus sequences: at least 15 passes were required for a consensus read to be considered for further analysis, and only individual base substitutions, deletions and insertions with high quality scores (QUAL = 93) were analyzed. The primer sites (40 bases) were excluded from error rate calculations. Additionally, to avoid potential sequencing and alignment artifacts, aligned reads were filtered for mapping quality (≥60), and alignments were required to span the entire reference sequence (starting within the 5′-primer region and ending in the 3′-primer region). Chimeric reads that mapped to more than one region in the reference sequence, as determined by BWA, or consensus reads with lengths deviating by more than 50 bases from the expected read length, were discarded.

**Figure 1.**
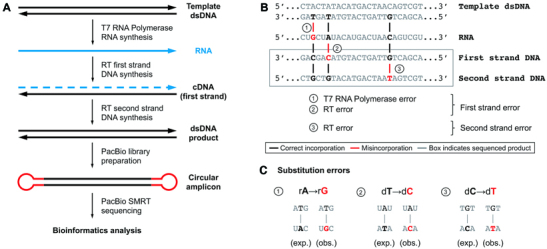
Measuring combined transcription and reverse transcription fidelity with PacBio sequencing. (**A**) Workflow. DNA templates are transcribed by T7 RNA polymerase with unmodified and modified NTPs to produce RNA. RNA is replicated by a reverse transcriptase to produce cDNA, then the first strand is replicated by the same reverse transcriptase to produce double-stranded DNA, which is then prepared for sequencing by ligating SMRTbell adaptors. (**B**) Identical first strand errors can arise by misincorporation from either the RNA polymerase or the reverse transcriptase (error types 1 and 2 in the figure, respectively). Only first strand errors confirmed in the second strand are counted. Second strand errors produce a mismatch between the first and second strand and represent misincorporation by the reverse transcriptase on DNA templates (error type 3 in the figure). (**C**) Substitution errors arising from misincorporation events. The first base is the expected, while the second is the observed base.

Error rates were derived as follows: first strand errors were determined by comparing cDNA to the reference sequence (RNA strand), and mutations were required to be present in both cDNA and the second-strand DNA (Figure 1). For second strand synthesis, errors were determined by comparing the second-strand to the first-strand DNA, and mutations were required to be present in the second strand but not in the first strand. The substitution, deletion and insertion error rates were calculated by dividing the respective number of mutations by the total number of bases for each sample. Deletions and insertions spanning multiple consecutive bases were counted as a single event as described in the results section. The average error and standard deviation were then calculated for each template and enzyme.

For comparing polymerase error rates and to estimate the statistical significance of observed differences, multiple measurements obtained on different templates (DNA-1, DNA-2, DNA-3 and DNA-4 and their modified version, where applicable) were compared using t-test at a significance value α = 0.05. When multiple comparisons were done, Bonferroni correction was applied to adjust the significance level according to the number of comparisons.

### Reverse transcriptase activity assay

Transcription templates were prepared by amplifying a DNA oligo containing a T7 promoter (5′-AATTAATACGACTCACTATAGAAGTATTTCTCCTCGCTGACTGAGATCGGAAGAGCACACGTCT-3′) with forward (5′-AATTAATACGACTCACTATAG-3′) and back (5′-AGACGTGTGCTCTTCCGATCT-3′) primers using Q5 High-Fidelity 2X Master Mix. PCR products were treated with Exonuclease III (*E. coli*) to degrade excess primers, cleaned using an Oligo DNA Clean & Concentrator (Zymo Research), and transcribed using the HiScribe T7 High Yield RNA Synthesis kit (20 μl total volume). After transcription, DNA was degraded by adding 4 U DNase I, 10 μl 10× DNase I Reaction Buffer in a 100 μl total volume and incubating for 30 min at 37°C. Transcription products were purified using a Zymo RNA Clean & Concentrator kit. Reverse transcriptase assays were performed by mixing 10 μl of enzyme mix (preheated for 1 min at 42°C) containing 0.026–0.26 pmol of reverse transcriptase, 1 mM each dNTP, 1× Reaction Buffer) and 10 μl of preheated substrate mix (1× Reaction buffer, 1 pmol RNA, 0.5 pmol FAM-labeled back primer (5′-/56-FAM/AGACGTGTGCTCTTCCGATCT-3′) and incubating at 42°C for 2 min. Reactions were quenched with 20 μl 20 mM EDTA, and analyzed using a 3730xl Genetic Analyzer (Applied Biosystems) and PeakScanner software as described in Greenough *et al*. (32).

## RESULTS

### Incorporation and reverse transcription of modified ribonucleotides

To address how modified bases affect polymerase fidelity, PacBio single-molecule sequencing was used to sequence double-stranded DNA synthesized from the reverse transcription of RNA and modified RNA (Figure 1). RNA templates were transcribed *in vitro* by T7 RNA polymerase. For modification studies, base-modified nucleotide triphosphates were incorporated in place of the equivalent unmodified base, producing RNA in which equivalent positions were replaced by the modified base (i.e. all adenosine substituted by *N*^6^-methyladenosine). Base composition analysis by LC–MS determined that the modified nucleotides were efficiently incorporated (Supplementary Table S1). However, compared to unmodified bases, the yield of full-length transcription products generally decreased with modified nucleotides (Supplementary Table S2). After transcription, the modified (or unmodified) RNA was reverse transcribed by either a reverse transcriptase (M-MuLV, AMV or ProtoScript II) or a DNA polymerase (*Bst* 2.0 or 3.0) to produce the first strand (cDNA). A second strand was synthesized by the same reverse transcriptase or DNA polymerase to produce double-stranded DNA, which was then sequenced. For analyzing cDNA errors, sequenced bases were compared to the reference to identify first strand errors, and only errors that were also confirmed in the second strand were counted. An error in the first strand can either be generated during transcription or reverse transcription, and first strand synthesis errors represent the cumulative errors of both T7 RNA polymerase and the reverse transcriptase. For example, if the expected RNA base was an A, but a C was observed in the cDNA, these substitutions would be classified as rA→rG/dT→dC, where the first pair represents the equivalent RNA polymerase error and the second pair represents the equivalent reverse transcriptase error that could have generated this substitution event (Figure 1B and C). However, errors uniquely arising in the second strand (i.e. producing mismatches between the first and second strand) can only be generated by the reverse transcriptase and were used to identify reverse transcriptase-specific errors on DNA templates. For comparing error rates, statistical analysis was performed as described in the methods.

**Figure 2.**
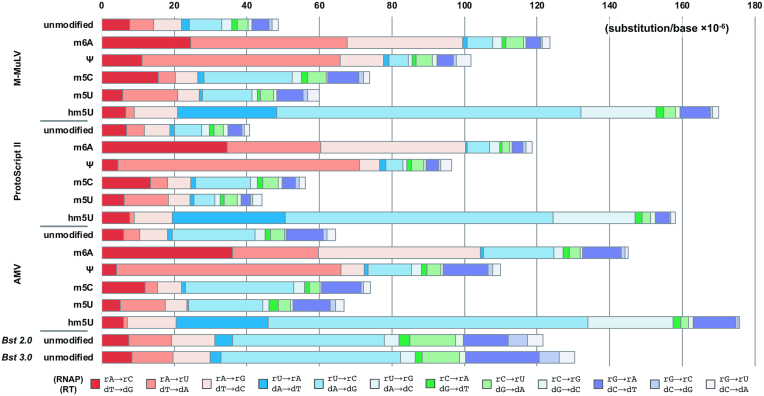
First strand (cDNA) synthesis error rates and error spectrum for unmodified and modified RNA. The RNA template is synthesized by T7 RNA polymerase, and then reverse transcribed by the reverse transcriptases shown in the figure. For comparison, also shown are the first strand error rate of *Bst* 2.0 and 3.0 DNA polymerases, DNA polymerases which can be used to reverse transcribe RNA. Polymerase substitution errors are written as the equivalent RNA polymerase substitution (top substitution; RNAP in the figure) or reverse transcriptase substitution (bottom substitution; RT in the figure).

When replicating unmodified RNA, all tested reverse transcriptases and DNA polymerases had first strand error rates ranging from 5.6 × 10^−5^ to 1.8 × 10^−4^ errors/base but the observed differences between the total error rates or the fraction of substitutions, insertions and deletions were not found to be statistically significant (Table 1). However, there was a statistically significant difference in the mutational spectrum of reverse transcriptases, with AMV reverse transcriptase displaying a higher preference for dA→dG substitutions over ProtoScript II and M-MuLV reverse transcriptases (Figure 2).

**Table 1.** Total error rates for cDNA strand synthesis of unmodified and modified RNA

		Percentage of total errors			
Template	Total error rate errors/base (× 10^−6^)	Substitution (%)	Deletion (%)	Insertion (%)	Total sequenced bases
*ProtoScript II Reverse Transcriptase and T7 RNA Polymerase*					
RNA	56 ± 8	71	19	10	30 868 961
m^6^A	152 ± 8	80	11	8	28 415 824
Ψ	101 ± 21	90	7	3	25 635 026
m^5^C	70 ± 4	82	12	6	25 784 603
m^5^U	54 ± 2	81	15	4	21 592 512
hm^5^U	188 ± 24	87	9	5	22 092 010
*M-MuLV Reverse Transcriptase and T7 RNA Polymerase*					
RNA	63 ± 12	78	11	11	16 815 378
m^6^A	149 ± 21	86	9	5	12 383 644
Ψ	114 ± 23	89	6	6	13 685 654
m^5^C	81 ± 18	86	9	5	18 210 833
m^5^U	65 ± 12	87	9	4	16 914 452
hm^5^U	185 ± 23	90	6	4	14 642 238
*AMV Reverse Transcriptase and T7 RNA Polymerase*					
RNA	75 ± 11	87	5	8	11 143 144
m^6^A	164 ± 11	89	5	6	11 313 430
Ψ	116 ± 22	94	4	3	14 114 144
m^5^C	81 ± 2	92	3	5	12 111 226
m^5^U	73 ± 5	91	5	3	9 918 107
hm^5^U	192 ± 8	91	5	4	14 633 922
*Bst 2.0 DNA Polymerase and T7 RNA Polymerase*					
RNA	179 ± 105	78	16	6	10 640 212
*Bst 3.0 DNA Polymerase and T7 RNA Polymerase*					
RNA	181 ± 102	82	15	4	13 459 274

Error rates for reverse transcription of RNA and modified RNA. First strand error rates are the combined error rates of T7 RNA polymerase and the reverse transcriptase described in the table. Total first strand error rates are an average of 4 different amplicons, with standard deviation reported between experiments. Distribution of substitution, deletion and insertion percentages of the total error rates are also shown.

**Figure 3.**
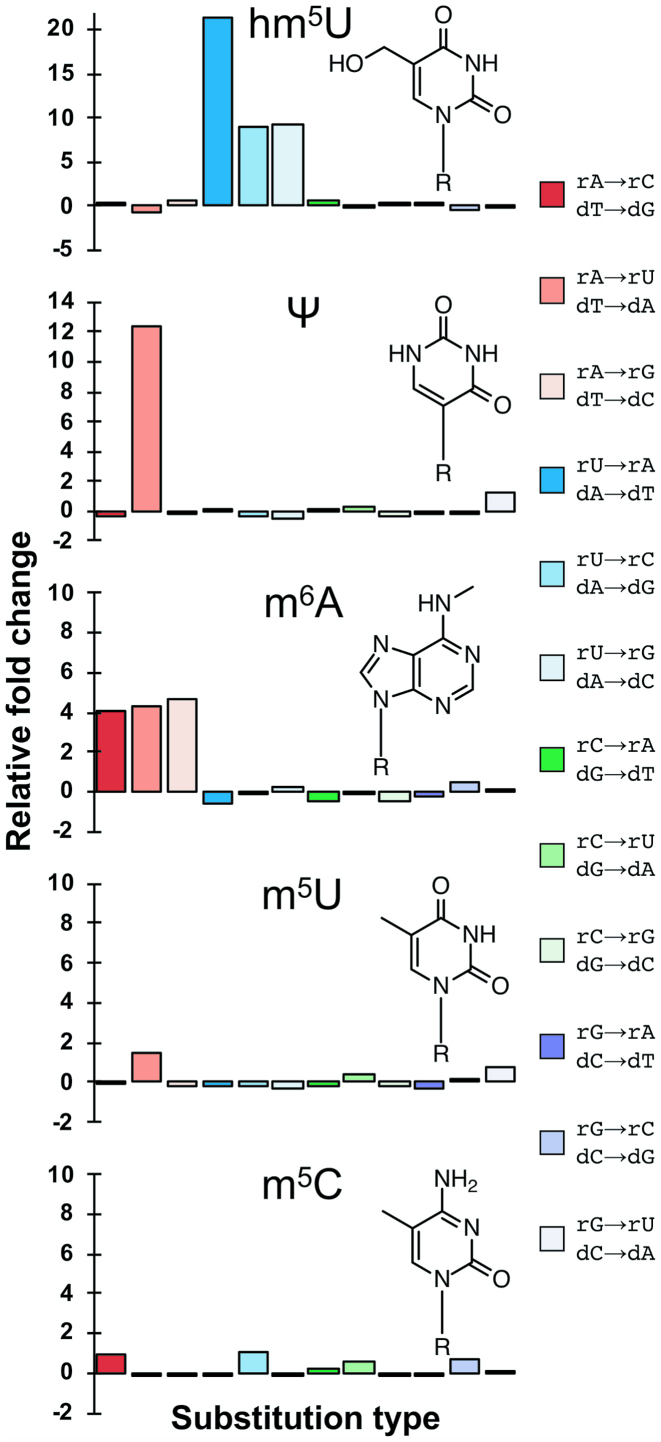
First strand error rates of modified RNA normalized to regular RNA (ProtoScript II reverse transcriptase). Relative substitution rates of each error type for each modification were normalized to regular RNA, for ProtoScript II reverse transcriptase (with T7 RNA polymerase). Relative fold change was calculated for each substitution type as (M – S)/S, where M is the substitution rate on RNA containing modified bases, and S is the substitution rate on unmodified RNA. A relative fold change of 0 represents no change in fidelity compared to unmodified RNA, whereas the numerical values represent the fold-change relative to unmodified RNA. For each non-reference error identified during cDNA synthesis, the equivalent RNA polymerase substitution (top pair) and reverse transcriptase substitution (bottom pair) that could generate the corresponding first strand error are identified.

The presence of modified nucleotides had differing effects on first strand errors. Pseudouridine (Ψ), hm^5^U and m^6^A had a statistically significant increase in the total first strand error rate compared to unmodified RNA, while m^5^C and m^5^U did not for all reverse transcriptases (Figure 2). To further study the effect of base modification on fidelity, first strand errors were normalized to unmodified RNA bases and Figure 3, Supplementary Figures S2 and S3 show the relative fold increase or decrease in error rates for each modification normalized to the unmodified base. For instance, *N*^6^-methyladenosine showed a 2- to 7-fold increase in the substitution events at adenosines (rA→rC/dT→dG, rA→rU/dT→dA, and rA→rG /dT→dC) indicating that *N*^6^-methyladenosine was more mutagenic than adenosine, by either increasing the mutation rate of reverse transcription or causing RNA polymerase misincorporation across dT. The increase in substitution events at adenosine positions was statistically significant for ProtoScript II and AMV reverse transcriptases. However, m^6^ATP was not misincorporated across other bases by T7 RNA polymerase, as there was no significant difference in substitution errors involving the incorporation of m^6^ATP (rU→rA, rC→rA, or rG→rA).

Of the uracil modifications tested, hm^5^U displayed the largest effect on first strand synthesis fidelity, by statistically significantly increasing substitutions at hm^5^U RNA reference positions by up to 21-fold (for dA→dT substitutions with ProtoScript II, Figure 3). These types of substitutions could be the result of either misincorporation by the reverse transcriptase across hm^5^U (resulting in dA→dT, dA→dG or dA→dC in DNA), or the RNA polymerase inserting the wrong nucleotide in place of hm^5^UTP (resulting in rU→rA, rU→rC or rU→rG substitutions in the RNA strand). In contrast, there was no statistically significant difference in errors involving RNA polymerase misincorporation of hm^5^UTP across non-complementary template bases (rA→rU, rC→rU, rG→rU for M-MuLV and AMV reverse transcriptases and rC→rU and rG→rU for ProtoScript II), indicating that when hm^5^UTP was incorporated into RNA, it was paired with template adenosine as frequently as UTP. Compared to hm^5^U, a smaller modification at the C5 position (m^5^U) also did not alter the fidelity of T7 RNA polymerase or reverse transcriptase, as almost all substitutions for m^5^U-substituted RNA showed no statistically significant difference between modified and unmodified uracil (the exceptions were rA→rU for M-MuLV and AMV reverse transcriptases). Base composition analysis of the RNA templates (Supplementary Table S1) showed the presence of uridine, likely due to UTP contamination of the synthetic nucleoside triphosphates, in both hm^5^U- (4%) and m^5^U- (11%) containing RNA samples. While this had little effect on the error rate for m^5^U-substituted RNA, it likely led to an underestimation of the overall misincorporation levels measured across hm^5^U.

Pseudouridine (Ψ) is an isomer of uridine where the base is attached by a carbon-carbon bond at the C5 position. Pseudouridine was misincorporated across thymidine by T7 RNA polymerase during transcription: rA→rU substitutions were increased 7- to 12-fold compared to unmodified RNA, while other substitutions (rG→rU or rC→rU) were unaffected (Figure 3, Supplementary Figures S2 and S3). There was a statistically significant difference between rA→rU error rate in pseudouridine-containing RNA when compared to respective error rates in unmodified RNA, and the difference was significant for all three reverse transcriptases (ProtoScript II, M-MuLV and AMV). Substitutions involving reverse transcription of template pseudouridine did not display a statistically significant difference when compared to uridine-containing RNA, indicating that pseudouridine was accurately replicated by M-MuLV, ProtoScript II and AMV reverse transcriptases.

### Sequence context analysis of first strand errors

**Figure 4.**
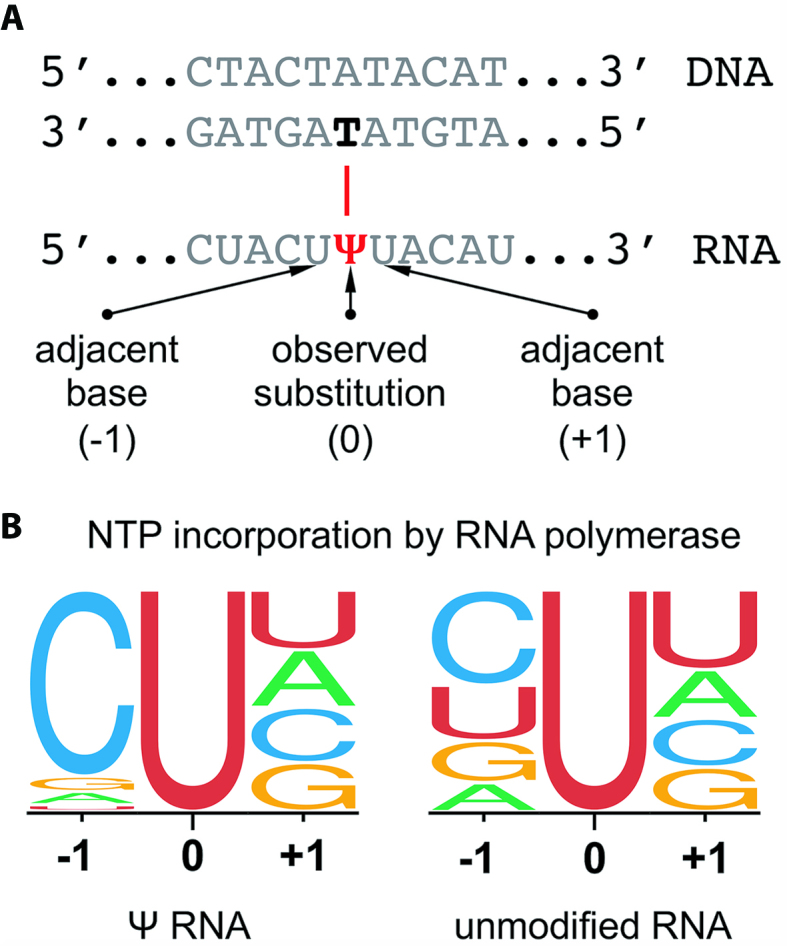
Sequence context analysis of first strand errors. (**A**) Sequence logos represent the identity of the bases surrounding each type of misincorporation, with respect to the reference RNA, (**B**) for pseudouridine-containing and unmodified RNA. In each logo, bases are ordered most frequently (top) to least frequently (bottom) observed. In this example, T7 RNA polymerase was used to generate the RNA template, and ProtoScript II reverse transcriptase was used for reverse transcription. A total of 1707 and 153 detected rA→rΨ and rA→rU substitutions, respectively, were analyzed (Supplementary Table S3).

To determine if replication errors were enriched in particular sequence contexts, the identity and frequency of the bases surrounding all first strand errors were analyzed. The artificial sequences used in the present study contained all possible 4-base combinations, ensuring an equimolar distribution of bases. However, for modified RNA bases, certain sequence contexts were enriched for substitution errors. For example, for pseudouridine, which mispairs with dT during transcription by T7 RNA polymerase, rA→rΨ substitutions were preferentially preceded by incorporation of CTP, a bias that is not seen with unmodified rA→rU substitutions (Figure 4). In Figure 4, a sequence logo represents the proportion of each base on either side of misincorporation events, written with respect to the synthesized RNA strand. A total of 1,707 rA→rΨ and 153 rA→rU substitution events were analyzed (Supplementary Table S3).

### Second strand synthesis error rates identify reverse transcriptase-specific error

**Figure 5.**
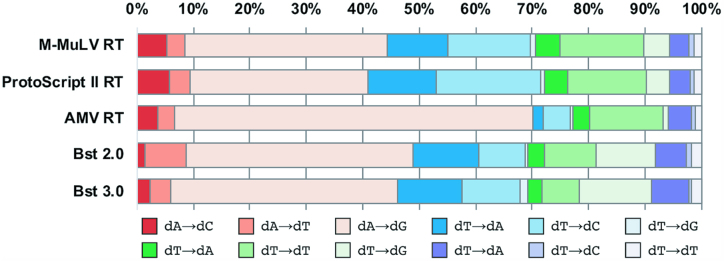
Normalized mutational spectrum of second strand error rates for reverse transcriptases or *Bst* DNA polymerases replicating DNA templates. Polymerase substitutions are written as (expected base) → (observed base).

Second strand errors reflect the isolated error rate of the reverse transcriptase on DNA templates and were identified by a mispair between the template base (first strand) and the incorporated base (second strand) (Figure 1). The total second strand error rates for M-MuLV, ProtoScript II and AMV reverse transcriptases, and *Bst* 2.0 and 3.0 DNA polymerases, were measured (Table 2), along with each polymerase mutational spectrum (Figure 5). The differences in error rates for M-MuLV, ProtoScript II and AMV reverse transcriptases were statistically significant. For all three reverse transcriptases and the *Bst* variants, the majority (approximately 70%) of all errors were at either adenosine or thymidine template bases, with the largest error class resulting from dA→dG substitutions. AMV reverse transcriptase had a statistically significant increase in dA→dG substitutions compared to M-MuLV and ProtoScript II reverse transcriptases. For isolated reverse transcriptase second strand errors, analysis of the bases surrounding substitution events indicated no discernible bias (Supplementary Figure S5).

**Table 2.** Total error rates for second strand synthesis

DNA polymerase	Total error rate (errors/base × 10^−6^)	Substitution	Deletion	Insertion	Total sequenced bases
ProtoScript II Reverse Transcriptase	62 ± 9	91%	6%	3%	154 391 090
M-MuLV Reverse Transcriptase	84 ± 19	92%	6%	3%	92 653 417
AMV Reverse Transcriptase	52 ± 4	93%	5%	2%	73 234 756
*Bst* 2.0 DNA Polymerase	62 ± 5	92%	7%	1%	10 640 457
*Bst* 3.0 DNA Polymerase	70 ± 23	89%	8%	3%	13 459 487

### Insertions and deletions

**Figure 6.**
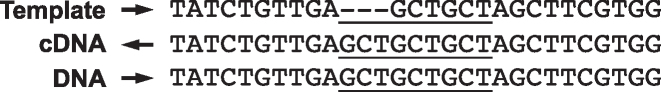
Notable 3-base insertion observed at position 219 in the reference sequence DNA-2. The first- and second-strand reads (mapped to the reference sequence, top) show a 3-base GCT insertion after a run of GCT repeats. Arrows indicate the direction of the original strand.

In addition to substitutions, insertion and deletion errors were also identified. Compared to the rate of base substitution, indels were relatively rare, representing <20% of the total errors by a particular polymerase (Table 1). Examining the length of observed deletions and insertions indicated that majority of them were 1 or 2 bases in length (Tables 3 and 4). One notable exception was observed during first strand synthesis of the template DNA-2, where a triplet insertion (GCT) was observed at position 219, sometimes as frequently as single base insertions (Figure 6). Notably, triplet insertions, but not deletions, were frequently observed. The triplet insertion occurred in a sequence context of GCT repeats and indicated polymerase slippage during replication of repetitive elements. Also, triplet insertions at position 219 were observed in first strand synthesis but not in second strand synthesis, indicating that the insertions were produced during first strand synthesis but faithfully replicated during second strand synthesis. Further examination of the positional distribution of insertions, deletions and substitutions in the first strand indicated that there were reference positions where errors were observed more frequently. For each error type (substitution, deletion and insertion), reference positions were ranked by error frequency, and the top three positions for each type were examined. These hotspot positions were almost invariably located in homopolymer, repetitive or structured regions. Supplementary Figure S6 provides the positional error maps for a few representative examples. Similar to first strand errors, second strand errors also had positional hot spots related to homopolymer and repetitive regions, however, the positions with frequently observed errors were largely different between the first and second strands.

**Table 3.** Size distribution of deletions and insertions for first strand synthesis

		Deletion size (nt) (%)				Insertion size (nt) (%)			
Enzyme	Template	1	2	3	≥4	1	2	3	≥4
ProtoScript II Reverse Transcriptase	RNA	56	28	2	14	45	5	42	8
	m^6^A	56	39	3	2	51	1	41	8
	Ψ	67	21	2	10	55	5	35	5
	m^5^C	40	27	2	31	69	5	23	3
	m^5^U	44	28	3	26	67	9	15	9
	hm^5^U	71	17	1	11	50	3	43	3
									
M-MuLV Reverse Transcriptase	RNA	49	38	5	9	42	5	50	3
	m^6^A	55	39	3	3	74	2	21	2
	Ψ	63	27	2	8	44	2	48	5
	m^5^C	52	26	1	21	58	1	36	4
	m^5^U	72	13	2	14	86	0	11	3
	hm^5^U	78	15	1	5	33	2	58	6
									
AMV Reverse Transcriptase	RNA	77	17	0	6	68	5	23	4
	m^6^A	81	15	2	2	58	2	31	9
	Ψ	69	23	4	4	68	5	24	3
	m^5^C	87	10	3	0	90	2	6	2
	m^5^U	82	18	0	0	88	0	8	4
	hm^5^U	87	10	1	1	33	4	59	4
									
*Bst* 2.0 DNA Polymerase	RNA	56	26	9	9	53	6	34	7
*Bst* 3.0 DNA Polymerase	RNA	57	29	9	5	46	8	32	13

The majority of deletions and insertions are 1 to 3 bases long. Overrepresentation of 3-base insertions are due to a triplet repeat element in the DNA-2 amplicon (Figure 6).

**Table 4.** Size distribution of deletions and insertions for second strand synthesis

		Deletion size (nt)%				Insertion size (nt) %			
Enzyme	Template	1	2	3	≥4	1	2	3	≥4
ProtoScript II Reverse Transcriptase	DNA	93	5	1	1	90	5	4	1
M-MuLV Reverse Transcriptase	DNA	90	6	1	3	85	9	4	2
AMV Reverse Transcriptase	DNA	95	5	0	0	86	8	3	3
*Bst* 2.0 DNA Polymerase	DNA	45	32	23	0	83	17	0	0
*Bst* 3.0 DNA Polymerase	DNA	69	21	8	1	71	5	10	14

Multi-base indels were considered a single slippage event in our study and as such were counted as a single insertion or deletion event, regardless of the number of bases inserted or deleted. Larger indels (four or more bases) occurred statistically more frequently in first strand synthesis than second strand synthesis, and large deletion events were more common than large insertions in the first strand. We additionally observed large-scale rearrangements in sequencing reads, where each rearrangement was a complex combination of multiple insertions, deletions and substitutions. These large-scale rearrangements were likely generated by template switching during transcription or cDNA synthesis and were excluded from error rate analysis by filtering for sequencing reads of the expected length.

### Replication efficiency of reverse transcriptases replicating modified bases

In addition to studying the accuracy of modified RNA, we measured the replication efficiency of substituted RNA by reverse transcriptases. By following replication of a 23-mer RNA template using a fluorescently labeled primer, the nucleotide incorporation turnover rate for each reverse transcriptase was determined (Table 5). Substituted RNA containing *N^6^*-methyladenosine was generally replicated less efficiently by ProtoScript II, M-MuLV and AMV reverse transcriptases. Template pseudouridine had no effect on the turnover rate of all three reverse transcriptases, nor did the methylated bases (m^5^C, m^5^U, hm^5^U) for ProtoScript II or m^5^C for AMV reverse transcriptases. Template hm^5^U was least efficiently replicated by ProtoScript II, reducing turnover by more than half that of unmodified RNA. Generally, with ProtoScript II, modifications that were accurately replicated did not affect reverse transcriptase specific activity, and modifications that decreased reverse transcriptase activity also decreased replication fidelity.

**Table 5.** Replication efficiency of reverse transcriptases on unmodified or modified RNA

Enzyme	Template	min^−1^	SD	Rel. to RNA
ProtoScript II Reverse Transcriptase	RNA	21	±3.9	1.00
	m^6^A	14	±2.4	0.66
	Ψ	22	±1.6	1.06
	m^5^C	18	±1.0	0.85
	m^5^U	20	±1.9	0.97
	hm^5^U	9	±1.2	0.44
				
M-MuLV Reverse Transcriptase	RNA	17	±1.1	1.00
	m^6^A	11	±0.4	0.63
	Ψ	17	±2.7	1.01
				
AMV Reverse Transcriptase	RNA	18	±1.1	1.00
	m^6^A	15	±1.2	0.86
	Ψ	24	±2.1	1.31
	m^5^C	20	±1.6	1.09

Reverse transcriptase turnover on short RNA, or RNA containing modified bases, and their relative efficiency compared to unmodified RNA. The turnover number (1/min) is the average of 3 independent reactions, with the standard deviation (SD) reported between replicates.

## DISCUSSION

Pacific Biosciences SMRT sequencing was used to measure the fidelity of incorporation and replication of modified and unmodified ribonucleotide bases. In this study, the effect of base modification was determined by normalizing the error rate for modified bases to unmodified RNA for each specific type of substitution. Seemingly small modifications, such as methyl-, hydroxymethyl- and isomeric bases, were found to increase combined RNA polymerase and reverse transcriptase (first strand) error rates.

Studies from DNA polymerases have shown how synthetic modified nucleotides have been used to probe the mechanism of polymerase fidelity (reviewed in (33,34)). DNA and RNA polymerases utilize a steric mechanism (one of several fidelity checkpoints) to ensure correct nucleotide incorporation and accurate genome replication (35). Similarly, the contribution of size and shape to RNA polymerase and reverse transcriptase fidelity has been probed using non-hydrogen bonding shape analogues (36,37). In this study, we utilize naturally-occurring nucleotides with small base modifications to probe the effect of different modifications on fidelity. For the C5 position of uracil, a small methyl group had minimal effect on RNA polymerase incorporation and reverse transcriptase replication fidelity. However, increasing the methyl group to add a larger polar hydroxyl group increased first strand errors. The C5 position is also frequently used as the site of attachment of fluorophores or affinity tags for derivatizing dU bases. Groups larger than a hydroxyl may have a greater effect on incorporation and replication fidelity. Indeed, bulky fluorophores reduce incorporation efficiency of wild-type DNA polymerases, and specialized polymerase variants have been created for synthesis of high density fluorescently-labeled DNA (38). The isomer pseudouridine, which contains a secondary amine at the equivalent C5 position in uracil, did not affect reverse transcriptase fidelity, but produced substitutions errors more frequently during RNA synthesis by T7 RNA polymerase. Misincorporation of pseudouridine by T7 RNA polymerase can have implications for RNA-based therapeutics, as pseudouridine is incorporated into RNA to reduce immunogenicity (6).

Reverse transcriptase-specific error rates on DNA templates were also determined by analyzing mismatches between the first and second strands. Results from this study can be compared to previously published error rates of reverse transcriptase acting on DNA templates using forward mutation assays. For AMV reverse transcriptase, we determined an error rate of 5.2 × 10^−5^ errors/base in this study, compared to 5.9 × 10^−5^ errors/base in Roberts *et al*. (15). However, there was a wider discrepancy for the error rate of M-MuLV reverse transcriptase, which was determined to be 8.4 × 10^−5^ errors/base in this study, compared to 3 × 10^−5^ errors/base previously determined using a forward mutation assay with DNA templates (15,17,39). It is also worth noting that although reverse transcriptase specific error rates could be determined on DNA templates in this study, reverse transcriptase fidelity on RNA templates could be different (16,19).

Polymerase slippage events, resulting in small insertions and deletions, were also characterized, and were shown to be a small percentage of the overall error rate for non-homopolymer sequence runs. The templates used in this study were artificial sequences designed to avoid homopolymers, which likely reduces observed error rates. In spite of the effort to reduce repetitive sequences, the artificial sequences still contained a particular trinucleotide repeat that was enriched for insertions of the triplet codon after cDNA synthesis. In contrast, this same repeat element did not increase triplet insertion for second strand synthesis, indicating that either T7 RNA polymerase was more prone to triplet insertions at this site during RNA synthesis or reverse transcriptases are more susceptible to insertions at this site when replicating RNA, but not DNA, templates. Both mechanisms are possible and would produce the same result in the current study.

Although reverse transcriptases are frequently utilized in RNA workflows, some DNA-dependent DNA polymerases have intrinsic activity on RNA templates (40). Utilizing a DNA polymerase for amplifying either RNA or DNA can be advantageous for simplifying molecular diagnostic assay and device development (41). In this study, we examined two engineered *Bst* DNA polymerase variants with innate reverse transcriptase activity (40,41) in order to compare their error rates and mutational spectrum to natural reverse transcriptases. Despite a lack of homology between Family A *Bst* DNA polymerase and viral reverse transcriptases, similar first strand error rates and mutational spectra were observed between the disparate polymerases. However, it should be noted that the relative contributions of RNA synthesis and RNA-templated reverse transcription to the overall first strand error rate cannot be determined in the current study and should be taken into account when interpreting results. Specifically, it is unclear whether T7 RNA polymerase and reverse transcriptases have similar or vastly different error rates, making it challenging to compare reverse transcriptase fidelity even on comparable RNA templates. Further characterization of polymerase fidelity by methods that can distinguish between RNA polymerase and reverse transcriptase errors is needed (25).

In conclusion, we developed a method to assess the relative accuracy of incorporation and replication of modified ribonucleotides and studied the effect of naturally-occurring RNA modifications on frequently used enzymes in biotechnology. Single-molecule next-generation sequencing assays provide comprehensive information on the frequency, type, and sequence context of replication errors and contribute to a better understanding of polymerase accuracy.

## DATA AVAILABILITY

Sequencing data pertaining to this study has been deposited into the Sequencing Read Archive under accession number SRP122531. Custom software tools are available in the GitHub repository at: https://github.com/potapovneb/rt-fidelity.

## Supplementary Material

Supplementary Data

## References

[1] Frye M., Jaffrey S.R., Pan T., Rechavi G., Suzuki T. RNA modifications: what have we learned and where are we headed. Nat. Rev. Genet. 2016; 17:365–372. 27140282 10.1038/nrg.2016.47

[2] Roundtree I.A., Evans M.E., Pan T., He C. Dynamic RNA modifications in gene expression regulation. Cell. 2017; 169:1187–1200. 28622506 10.1016/j.cell.2017.05.045 PMC5657247

[3] Cantara W.A., Crain P.F., Rozenski J., McCloskey J.A., Harris K.A., Zhang X., Vendeix F.A.P., Fabris D., Agris P.F. The RNA modification database, RNAMDB: 2011 update. Nucleic Acids Res. 2011; 39:D195–D201. 21071406 10.1093/nar/gkq1028 PMC3013656

[4] Gilbert W.V., Bell T.A., Schaening C. Messenger RNA modifications: form, distribution, and function. Science. 2016; 352:1408–1412. 27313037 10.1126/science.aad8711 PMC5094196

[5] Song J., Yi C. Chemical modifications to RNA: a new layer of gene expression regulation. ACS Chem. Biol. 2017; 12:316–325. 28051309 10.1021/acschembio.6b00960

[6] Karikó K., Muramatsu H., Welsh F.A., Ludwig J., Kato H., Akira S., Weissman D. Incorporation of pseudouridine into mRNA yields superior nonimmunogenic vector with increased translational capacity and biological stability. Mol. Ther. 2008; 16:1833–1840. 18797453 10.1038/mt.2008.200 PMC2775451

[7] Dominissini D., Moshitch-Moshkovitz S., Schwartz S., Salmon-Divon M., Ungar L., Osenberg S., Cesarkas K., Jacob-Hirsch J., Amariglio N., Kupiec M. Topology of the human and mouse m6A RNA methylomes revealed by m6A-seq. Nature. 2012; 485:201–206. 22575960 10.1038/nature11112

[8] Meyer K.D., Saletore Y., Zumbo P., Elemento O., Mason C.E., Jaffrey S.R. Comprehensive analysis of mRNA methylation reveals enrichment in 3′ UTRs and near stop codons. Cell. 2012; 149:1635–1646. 22608085 10.1016/j.cell.2012.05.003 PMC3383396

[9] Squires J.E., Patel H.R., Nousch M., Sibbritt T., Humphreys D.T., Parker B.J., Suter C.M., Preiss T. Widespread occurrence of 5-methylcytosine in human coding and non-coding RNA. Nucleic Acids Res. 2012; 40:5023–5033. 22344696 10.1093/nar/gks144 PMC3367185

[10] Carlile T.M., Rojas-Duran M.F., Zinshteyn B., Shin H., Bartoli K.M., Gilbert W.V. Pseudouridine profiling reveals regulated mRNA pseudouridylation in yeast and human cells. Nature. 2014; 515:143–146. 25192136 10.1038/nature13802 PMC4224642

[11] Schwartz S., Bernstein D.A., Mumbach M.R., Jovanovic M., Herbst R.H., León-Ricardo B.X., Engreitz J.M., Guttman M., Satija R., Lander E.S. Transcriptome-wide mapping reveals Widespread Dynamic-Regulated pseudouridylation of ncRNA and mRNA. Cell. 2014; 159:148–162. 25219674 10.1016/j.cell.2014.08.028 PMC4180118

[12] Linder B., Grozhik A.V., Olarerin-George A.O., Meydan C., Mason C.E., Jaffrey S.R. Single-nucleotide-resolution mapping of m6A and m6Am throughout the transcriptome. Nat. Methods. 2015; 12:767–772. 26121403 10.1038/nmeth.3453 PMC4487409

[13] Li X., Xiong X., Yi C. Epitranscriptome sequencing technologies: decoding RNA modifications. Nat. Methods. 2016; 14:23–31. 28032622 10.1038/nmeth.4110

[14] Preston B., Poiesz B., Loeb L. Fidelity of HIV-1 reverse transcriptase. Science. 1988; 242:1168–1171. 2460924 10.1126/science.2460924

[15] Roberts J.D., Preston B.D., Johnston L.A., Soni A., Loeb L.A., Kunkel T.A. Fidelity of two retroviral reverse transcriptases during DNA-dependent DNA synthesis in vitro. Mol. Cell. Biol. 1989; 9:469–476. 2469002 10.1128/mcb.9.2.469-476.1989 PMC362622

[16] Boyer J.C., Bebenek K., Kunkel T.A. Unequal human immunodeficiency virus type 1 reverse transcriptase error rates with RNA and DNA templates. Proc. Natl. Acad. Sci. U.S.A. 1992; 89:6919–6923. 1379727 10.1073/pnas.89.15.6919 PMC49616

[17] Ji J., Loeb L.A. Fidelity of HIV-1 reverse transcriptase copying RNA in vitro. Biochemistry. 1992; 31:954–958. 1370910 10.1021/bi00119a002

[18] Kati W.M., Johnson K.A., Jerva L.F., Anderson K.S. Mechanism and fidelity of HIV reverse transcriptase. J. Biol. Chem. 1992; 267:25988–25997. 1281479

[19] Yu H., Goodman M.F. Comparison of HIV-1 and avian myeloblastosis virus reverse transcriptase fidelity on RNA and DNA templates. J. Biol. Chem. 1992; 267:10888–10896. 1375233

[20] Menéndez-Arias L. Mutation rates and intrinsic fidelity of retroviral reverse transcriptases. Viruses. 2009; 1:1137–1165. 21994586 10.3390/v1031137 PMC3185545

[21] Imashimizu M., Oshima T., Lubkowska L., Kashlev M. Direct assessment of transcription fidelity by high-resolution RNA sequencing. Nucleic Acids Res. 2013; 41:9090–9104. 23925128 10.1093/nar/gkt698 PMC3799451

[22] Yasukawa K., Iida K., Okano H., Hidese R., Baba M., Yanagihara I., Kojima K., Takita T., Fujiwara S. Next-generation sequencing-based analysis of reverse transcriptase fidelity. Biochem. Biophys. Res. Commun. 2017; 492:147–153. 28778390 10.1016/j.bbrc.2017.07.169

[23] Álvarez M., Barrioluengo V., Afonso-Lehmann R.N., Menéndez-Arias L. Altered error specificity of RNase H-deficient HIV-1 reverse transcriptases during DNA-dependent DNA synthesis. Nucleic Acids Res. 2013; 41:4601–4612. 23444139 10.1093/nar/gkt109 PMC3632107

[24] Gout J.-F., Thomas W.K., Smith Z., Okamoto K., Lynch M. Large-scale detection of in vivo transcription errors. Proc. Natl. Acad. Sci. U.S.A. 2013; 110:18584–18589. 24167253 10.1073/pnas.1309843110 PMC3832031

[25] Reid-Bayliss K.S., Loeb L.A. Accurate RNA consensus sequencing for high-fidelity detection of transcriptional mutagenesis-induced epimutations. Proc. Natl. Acad. Sci. U.S.A. 2017; 114:9415–9420. 28798064 10.1073/pnas.1709166114 PMC5584456

[26] Gout J.-F., Li W., Fritsch C., Li A., Haroon S., Singh L., Hua D., Fazelinia H., Smith Z., Seeholzer S. The landscape of transcription errors in eukaryotic cells. Sci. Adv. 2017; 3:e1701484. 29062891 10.1126/sciadv.1701484 PMC5650487

[27] Hauenschild R., Tserovski L., Schmid K., Thüring K., Winz M.-L., Sharma S., Entian K.-D., Wacheul L., Lafontaine D.L.J., Anderson J. The reverse transcription signature of N-1-methyladenosine in RNA-Seq is sequence dependent. Nucleic Acids Res. 2015; 43:9950–9964. 26365242 10.1093/nar/gkv895 PMC4787781

[28] Alenko A., Fleming A.M., Burrows C.J. Reverse transcription past products of guanine oxidation in RNA Leads to insertion of a and c opposite 8-Oxo-7,8-dihydroguanine and A and G opposite 5-Guanidinohydantoin and Spiroiminodihydantoin Diastereomers. Biochemistry. 2017; 56:5053–5064. 28845978 10.1021/acs.biochem.7b00730 PMC5623583

[29] Sooknanan R., Howes M., Read L., Malek L.T. Fidelity of nucleic acid amplification with avian myeloblastosis virus reverse transcriptase and T7 RNA polymerase. BioTechniques. 1994; 17:1077–1080. 7532977

[30] Potapov V., Ong J.L. Examining sources of error in PCR by single-molecule sequencing. PLoS ONE. 2017; 12:e0169774. 28060945 10.1371/journal.pone.0169774 PMC5218489

[31] Li H., Durbin R. Fast and accurate long-read alignment with Burrows-Wheeler transform. Bioinformatics. 2010; 26:589–595. 20080505 10.1093/bioinformatics/btp698 PMC2828108

[32] Greenough L., Schermerhorn K.M., Mazzola L., Bybee J., Rivizzigno D., Cantin E., Slatko B.E., Gardner A.F. Adapting capillary gel electrophoresis as a sensitive, high-throughput method to accelerate characterization of nucleic acid metabolic enzymes. Nucleic Acids Res. 2016; 44:e15. 26365239 10.1093/nar/gkv899 PMC4737176

[33] Lee I., Berdis A.J. Non-natural nucleotides as probes for the mechanism and fidelity of DNA polymerases. Biochim. Biophys. Acta. 2010; 1804:1064–1080. 19733263 10.1016/j.bbapap.2009.08.023 PMC3149816

[34] Kool E.T. Active site tightness and substrate fit in DNA replication. Annu. Rev. Biochem. 2002; 71:191–219. 12045095 10.1146/annurev.biochem.71.110601.135453

[35] Johnson S.J., Beese L.S. Structures of mismatch replication errors observed in a DNA polymerase. Cell. 2004; 116:803–816. 15035983 10.1016/s0092-8674(04)00252-1

[36] Ulrich S., Kool E.T. Importance of steric effects on the efficiency and fidelity of transcription by T7 RNA polymerase. Biochemistry. 2011; 50:10343–10349. 22044042 10.1021/bi2011465 PMC3222776

[37] Silverman A.P., Garforth S.J., Prasad V.R., Kool E.T. Probing the active site steric flexibility of HIV-1 reverse transcriptase: different constraints for DNA- versus RNA-templated synthesis. Biochemistry. 2008; 47:4800–4807. 18366188 10.1021/bi702427y PMC4160155

[38] Ramsay N., Jemth A.-S., Brown A., Crampton N., Dear P., Holliger P. CyDNA: Synthesis and Replication of Highly Cy-Dye Substituted DNA by an Evolved Polymerase. J. Am. Chem. Soc. 2010; 132:5096–5104. 20235594 10.1021/ja909180c PMC2850551

[39] Roberts J.D., Bebenek K., Kunkel T.A. The accuracy of reverse transcriptase from HIV-1. Science. 1988; 242:1171–1173. 2460925 10.1126/science.2460925

[40] Shi C., Shen X., Niu S., Ma C. Innate reverse transcriptase activity of DNA polymerase for isothermal RNA direct detection. J. Am. Chem. Soc. 2015; 137:13804–13806. 26474356 10.1021/jacs.5b08144

[41] Lee D., Shin Y., Chung S., Hwang K.S., Yoon D.S., Lee J.H. Simple and highly sensitive molecular diagnosis of Zika Virus by lateral flow assays. Anal. Chem. 2016; 88:12272–12278. 28193014 10.1021/acs.analchem.6b03460

