# Supplementary material for: Base modifications affecting RNA polymerase and reverse transcriptase fidelity
Source: Nucleic Acids Res. 2018 May 10;46(11):5753–63. doi: 10.1093/nar/gky341 (PMC6009661; doi:10.1093/nar/gky341)
Supplement: Supplementary Data [file gky341_supplemental_figures.pdf]

Base Modifications Affecting RNA Polymerase and Reverse Transcriptase Fidelity

SUPPLEMENTARY DATA

Table S1. Base composition of each RNA base detected by LC-MS, relative to guanosine.

Table S2. Relative yield of T7 RNA polymerase synthesis of modified RNA, normalized to unmodified RNA.

Table S3. Total number of substitutions, deletions, and insertions for cDNA strand synthesis of unmodified and modified RNA.

Table S4. Total number of substitutions, deletions, and insertions for second strand synthesis.

Table S5. Length and base composition of template sequences.

Figure S1. Modified RNA base composition.

Figure S2. First strand error rates of modified RNA normalized to unmodified RNA (M-MuLV reverse transcriptase).

Figure S3. First strand error rates of modified RNA normalized to unmodified RNA (AMV reverse transcriptase).

Figure S4. Sequence context of first strand errors.

Figure S5. Sequence context of second strand errors from ProtoScript II reverse transcriptase.

Figure S6. Example of hot spot positions for indels and substitutions.

Data S1. Template sequences

Data S2. Oligonucleotide sequences

Table S1. Base composition of each RNA base detected by LC-MS, relative to guanosine.

Transcription product	A	m^6A	U	Ψ	m^5U	hm^5U	C	m^5C	G
RNA	0.99 ± 0.02		1.05 ± 0.01				0.98 ± 0.01		1
m^6A	0.03 ± 0.01	1.02 ± 0.01	1.05 ± 0.01				0.98 ± 0.01		1
Ψ	0.98 ± 0.01			0.99 ± 0.01			0.98 ± 0.01		1
m^5C	0.94 ± 0.10		0.99 ± 0.11					0.95 ± 0.01	1
m^5U	1.01 ± 0.02		0.11 ± 0.02		1.04 ± 0.02		1.15 ± 0.02		1
hm^5U	1.01 ± 0.06		0.04 ± 0.01			0.92 ± 0.01	0.96 ± 0.01		1

Measurements are an average of N = 4 different transcription products used for fidelity studies. The relative abundance of each nucleoside was determined by dividing the UV absorbance by the corresponding extinction coefficient and normalized to G (set as 1). For m^5U-substituted RNA, the relative abundance of each nucleoside was determined by LC-MS/MS.

Methods. Incorporation efficiency of modified nucleotides was assessed by Liquid Chromatography-Mass Spectrometry (LC-MS). RNA samples (1 µg) were digested to nucleosides by incubation with 1 µL of the Nucleoside Digestion Mix (New England Biolabs) in 20 µL of 1X Nucleoside Digestion Mix Reaction Buffer at 37 °C for 1 h. Digested RNA samples were directly analyzed by LC-MS without purification. LC-MS analysis was performed on an Agilent 1200 Series LC/MS System equipped with a G1315D diode array detector and a 6120 Single Quadrupole Mass Detector in both positive (+ESI) and negative (-ESI) electrospray ionization modes. LC was performed on a Waters Atlantis T3 column (4.6 × 150 mm, 3 µm) with a gradient mobile phase consisting of aqueous ammonium acetate (10 mM, pH 4.5) and methanol. The relative abundance of each nucleoside was determined by UV absorbance and normalized relative to guanosine. LC-MS/MS was performed on an Agilent 1290 UHPLC equipped with a G4212A diode array detector and a 6490A Triple Quadrupole Mass Detector operating under positive electrospray ionization mode (+ESI). UHPLC was performed using a Waters XSelect HSS T3 XP column (2.1 × 100 mm, 2.5 µm) with a gradient mobile phase consisting of aqueous ammonium formate (10 mM, pH 4.4) and methanol. MS data acquisition was performed in the dynamic multiple reaction monitoring (DMRM) mode. The relative abundance of each nucleoside was also normalized relative to guanosine.

Table S2. Relative yield of T7 RNA polymerase synthesis of modified RNA, normalized to unmodified RNA.

Modification	Relative transcription yield, %
unmodified NTPs	100 ± 0
m^6A	31 ± 7
Ψ	26 ± 18
m^5C	67 ± 15
m^5U	73 ± 12
hm^5U	17 ± 4

Table S3. Total number of substitutions, deletions, and insertions for cDNA strand synthesis of unmodified and modified RNA.

Template	Substitutions	Deletions	Insertions	rA→rC dT→dG	rA→rU dT→dA	rA→rG dT→dC	rU→rA dA→dT	rU→rC dA→dG	rU→rG dA→dC	rC→rA dG→dT	rC→rU dG→dA	rC→rG dG→dC	rG→rA dC→dT	rG→rC dC→dG	rG→rU dC→dA
ProtoScript II Reverse Transcriptase and T7 RNA Polymerase															
RNA	1256	379	191	212	153	215	43	227	69	38	81	36	117	20	45
m6A	3373	738	334	981	738	1130	14	177	76	19	62	16	85	27	48
Ψ	2475	319	80	115	1707	143	42	122	27	33	86	19	84	16	81
m5C	1445	275	101	347	123	166	32	387	54	38	108	27	96	28	39
m5U	953	196	46	135	263	129	23	122	34	22	80	20	56	14	55
hm5U	3493	400	207	173	25	234	687	1629	500	41	52	29	84	8	31
M-MuLV Reverse Transcriptase and T7 RNA Polymerase															
RNA	818	103	153	130	113	126	36	152	45	25	51	18	78	16	28
m6A	1530	179	84	305	534	392	17	86	33	12	61	9	48	7	26
Ψ	1392	129	81	152	747	164	22	73	13	15	61	18	62	11	54
m5C	1346	117	69	284	85	115	31	440	49	29	94	10	153	23	33
m5U	1016	88	37	98	255	102	14	232	24	14	63	16	122	19	57
hm5U	2489	143	144	99	32	176	401	1226	303	32	47	17	123	9	24
AMV Reverse Transcriptase and T7 RNA Polymerase															
RNA	719	53	57	66	52	84	15	254	30	18	42	5	114	11	28
m6A	1642	108	95	408	268	505	10	218	30	18	34	7	122	11	11
Ψ	1551	71	38	57	875	89	16	167	40	21	54	8	176	17	31
m5C	896	39	48	146	41	79	13	361	38	15	37	4	131	10	21
m5U	662	34	25	51	123	60	4	203	17	24	35	6	102	14	23
hm5U	2573	148	122	89	16	196	371	1290	342	31	30	21	172	7	8
Bst 2.0 DNA Polymerase and T7 RNA Polymerase															
RNA	1295	254	98	81	123	127	53	446	43	31	133	24	130	57	47
Bst 3.0 DNA Polymerase and T7 RNA Polymerase															
RNA	1755	301	84	112	154	138	40	664	54	28	137	24	271	76	57

Table S4. Total number of substitutions, deletions, and insertions for second strand synthesis.

Enzyme	Substitutions	Deletions	Insertions	dA→dC	dA→dT	dA→dG	dT→dA	dT→dC	dT→dG	dT→dA	dT→dT	dT→dG	dT→dA	dT→dC	dT→dT
ProtoScript II RT	8855	603	177	508	328	2776	1074	1646	57	362	1236	365	326	65	112
M-MuLV RT	6742	380	172	359	218	2418	718	990	58	291	992	312	226	72	88
AMV RT	3586	210	72	136	105	2276	62	175	11	108	469	33	142	25	44
Bst 2.0 DNA Pol	602	44	6	9	44	241	71	49	2	18	55	64	33	5	11
Bst 3.0 DNA Pol	779	71	21	18	28	314	88	81	10	19	52	99	52	4	14

Table S5. Length and base composition of template sequences.

Template	Length, nt	A, nt (%)	C, nt (%)	G, nt (%)	T, nt (%)
DNA-1	1076	267 (24.8%)	271 (25.2%)	269 (25.0%)	269 (25.0%)
DNA-2	1078	268 (24.9%)	271 (25.1%)	269 (25.0%)	270 (25.0%)
DNA-3	564	140 (24.8%)	144 (25.5%)	142 (25.2%)	138 (24.5%)
DNA-4	552	137 (24.8%)	141 (25.5%)	139 (25.2%)	135 (24.5%)

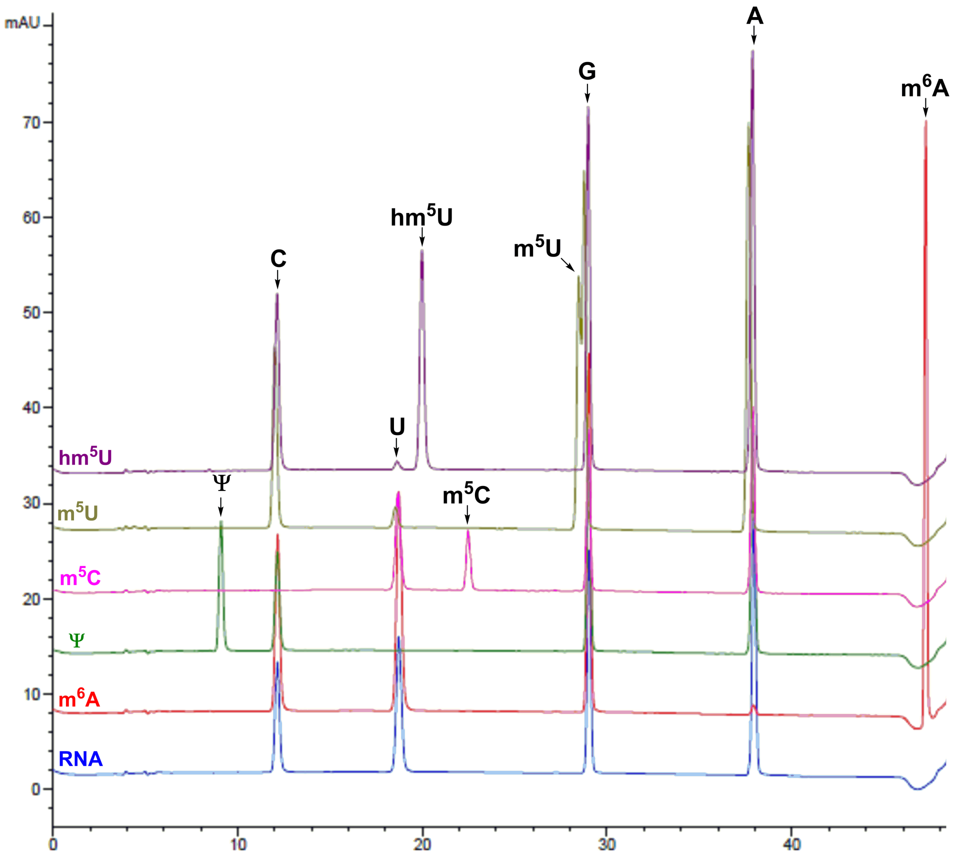

Figure S1. Modified RNA base composition. LC-MS base composition analysis of modified and unmodified RNA. Representative LC-MS traces (arbitrary units over minutes) with peak ID's for unmodified RNA (blue), and RNA fully substituted with m^{6}A (red), Ψ (green), m^{5}C (pink), m^{5}U (yellow), hm^{5}U (purple).

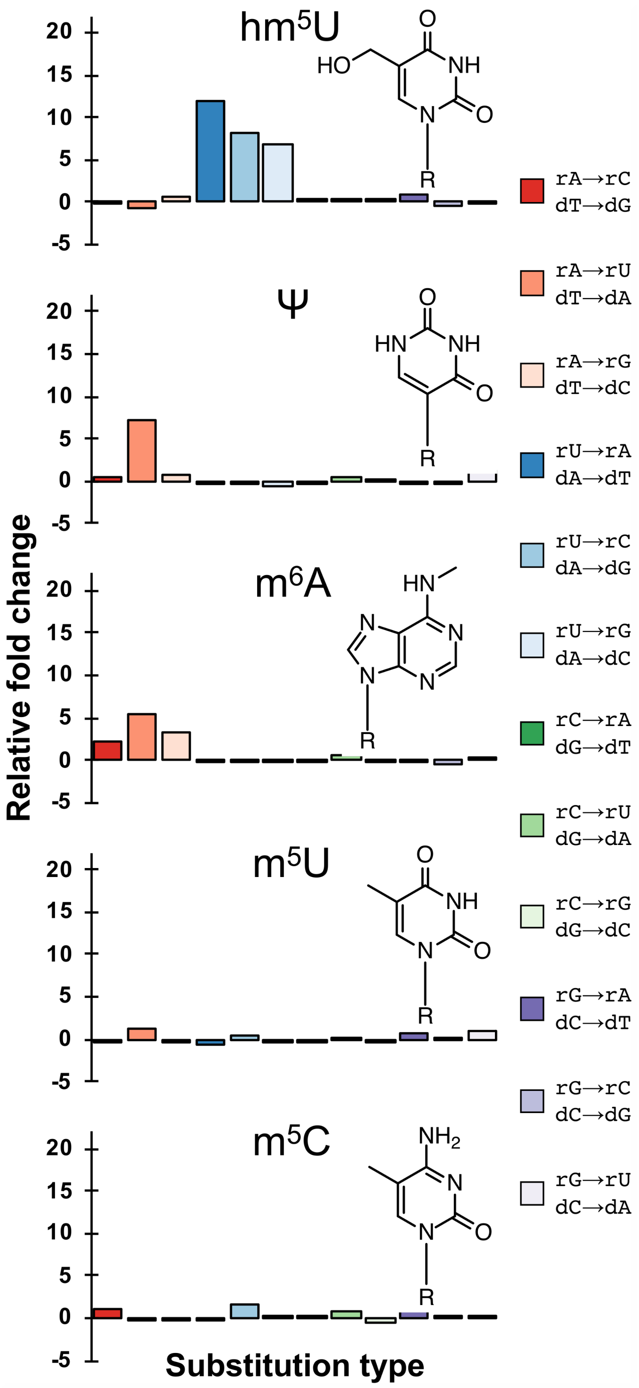

Figure S2. First strand error rates of modified RNA normalized to unmodified RNA (M-MuLV reverse transcriptase). Relative substitution rates of each error type for each modification were normalized to regular RNA, for M-MuLV reverse transcriptase (with T7 RNA polymerase). Relative fold change was calculated for each substitution type as (M – S) / S, where M is the substitution rate on RNA containing modified bases, and S is the substitution rate on the unmodified RNA. A relative fold change of 0 represents no change in fidelity compared to unmodified RNA, whereas the numerical values represent the fold-change relative to unmodified RNA. For each non-reference error identified during cDNA synthesis, the equivalent RNA polymerase substitution (top pair) and reverse transcriptase substitution (bottom pair) that could generate the corresponding first strand error are identified.

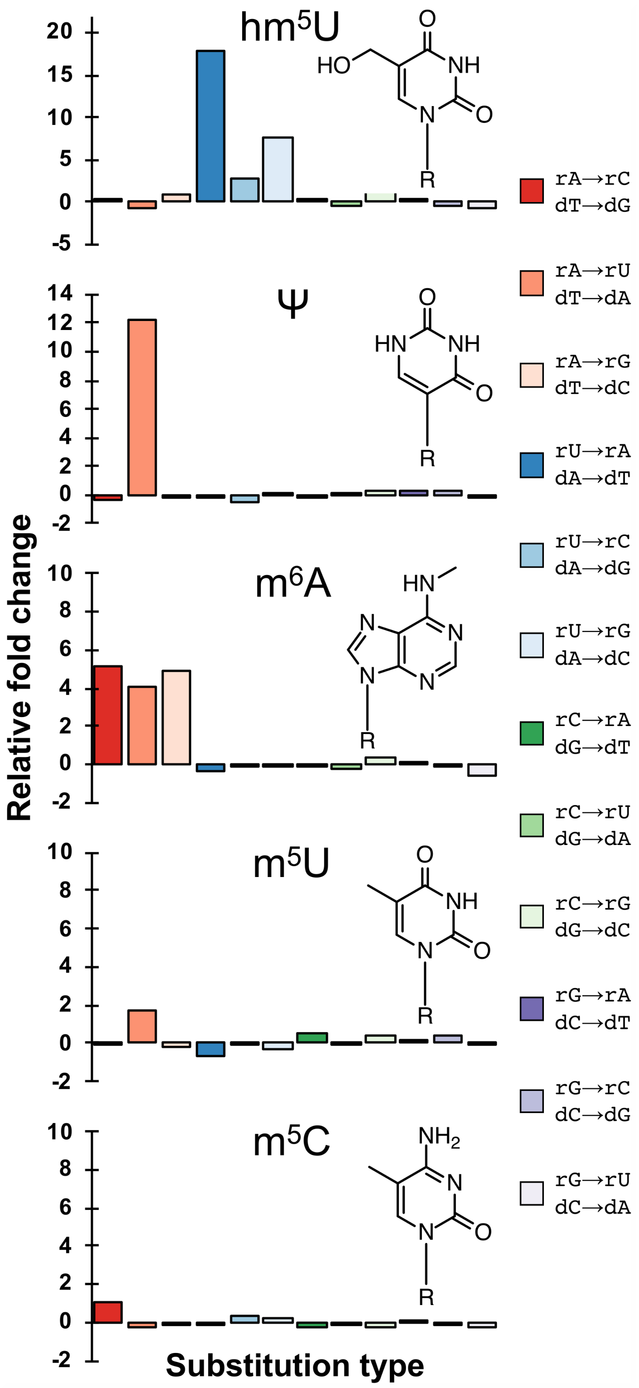

Figure S3. First strand error rates of modified RNA normalized to unmodified RNA (AMV reverse transcriptase). Relative substitution rates of each error type for each modification were normalized to regular RNA, for AMV reverse transcriptase (with T7 RNA polymerase). Relative fold change was calculated for each substitution type as (M – S) / S, where M is the substitution rate on RNA containing modified bases, and S is the substitution rate on the unmodified RNA. A relative fold change of 0 represents no change in fidelity compared to unmodified RNA, whereas the numerical values represent the fold-change relative to unmodified RNA. For each non-reference error identified during cDNA synthesis, the equivalent RNA polymerase substitution (top pair) and reverse transcriptase substitution (bottom pair) that could generate the corresponding first strand error are identified.

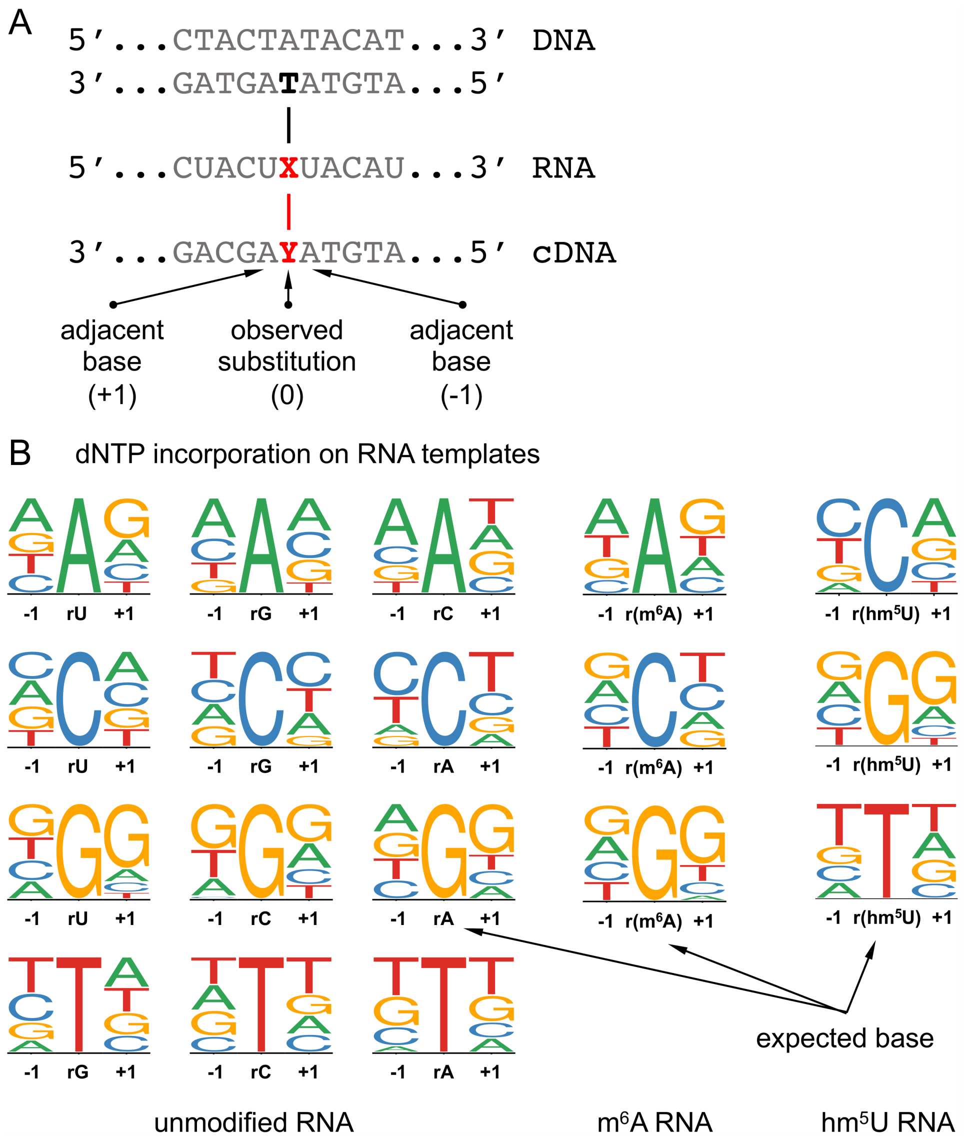

Figure S4. Sequence context of first strand errors. (A) Schematics of sequence context analysis of first strand errors. For each substitution type, the distribution of bases incorporated before and after the substitution event was analyzed. (B) Sequence logos represent the frequency and identity of the bases surrounding each substitution type, with respect to the first strand cDNA, for standard RNA, m^{6}A- and hm^{5}U-containing RNA templates. In each logo, bases are ordered most frequently (top) to least frequently (bottom) observed. For modified RNA templates, only the major type of substitution events are shown. In this example, T7 RNA polymerase was used to generate the RNA template, and ProtoScript II reverse transcriptase was the reverse transcriptase.

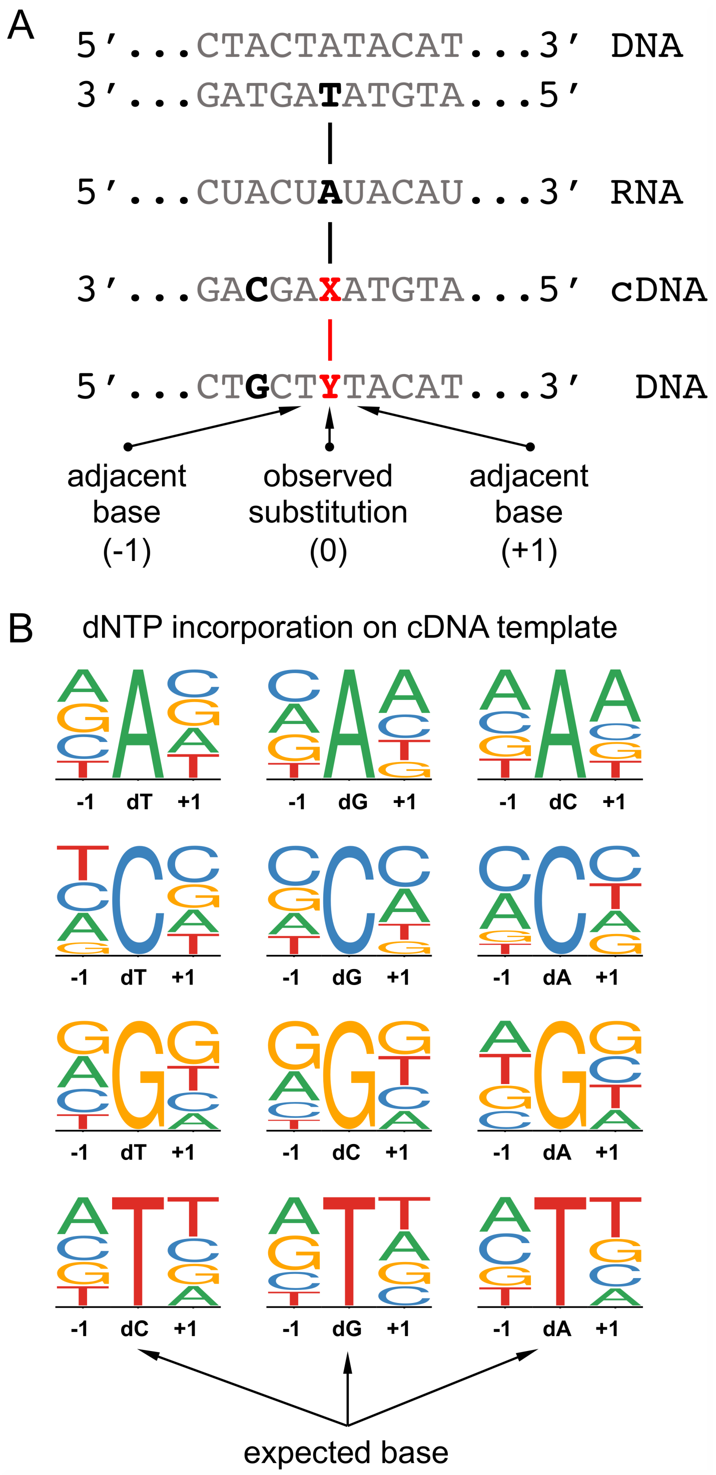

Figure S5. Sequence context of second strand errors from ProtoScript II reverse transcriptase. (A) Schematics of sequence context analysis of second strand errors, which represent reverse transcriptase errors only. For each substitution type, the distribution of bases incorporated before and after the substitution event was analyzed. (B) Sequence logos represent the frequency and identity of the bases surrounding each type of substitution, with respect to second strand cDNA. In each logo, bases are ordered most frequently (top) to least frequently (bottom) observed.

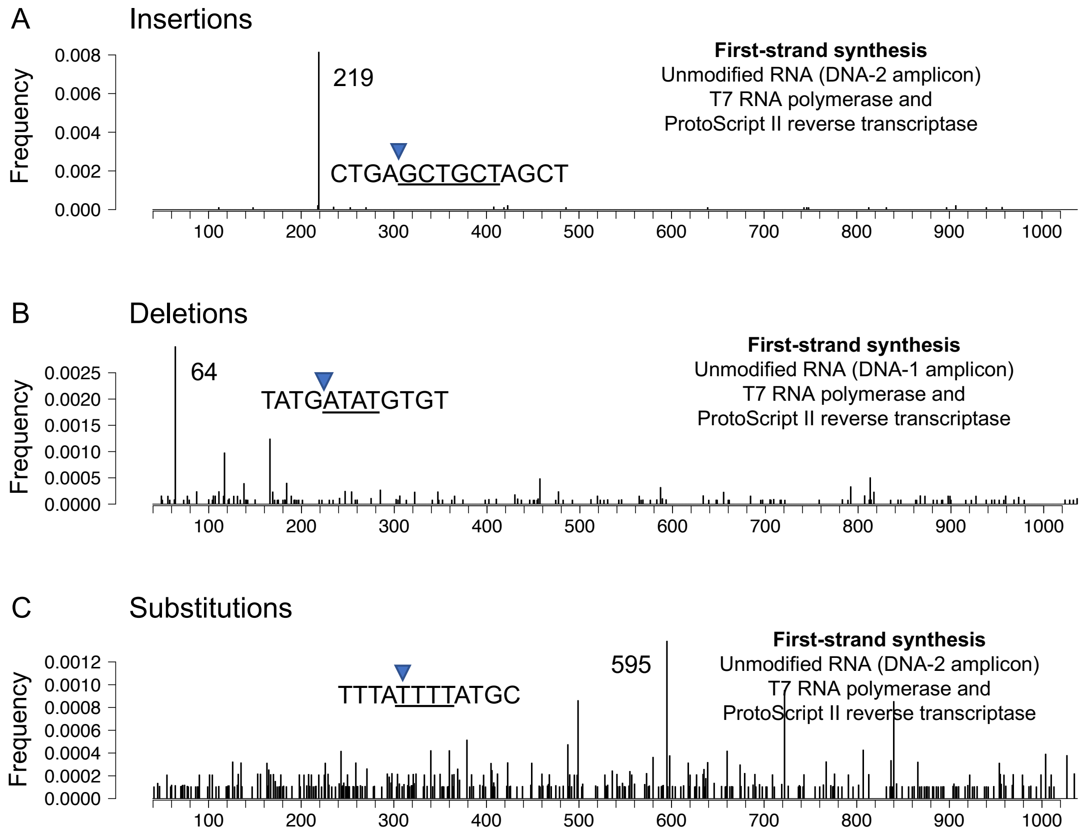

Figure S6. Example of hot spot positions for indels and substitutions. (A) A 3-base insertion GCT is frequently observed at position 219 immediately adjacent to GCT repeat in template sequence. (B) A 2-base deletion AT is frequently observed at position 64. (C) The first position in homopolymer regions is a hot spot for substitutions. For each error type (substitution, deletion and insertion) positions were ranked by error frequency, and we focused on top three positions for each type.

Data S1. Template sequences

```
>DNA-1
ACGAGTCAGGCTACAGCATCCTCTGGTTCAGACTACTTGATTCATGTGTACCCTATATGC
GAGGATATGTGTATCGTAGAAATTGTCAGGCAGTAACGTTCCGCGAGTTTTAATGGGCGC
GCCATGACTCTAAGAGTGATATACCTCCTCGGTCTCGGGCCCGGGGTGTAATTAGCCCAG
TTAGACACGATCGCCCGACGTATATTGTTGCTTGGGTATCGTCGCATGCGAAGTATTGCC
CAAGGAGACACAACAAGCAACTTATGTTGACTCCCTTCGACCATTAAAATTTGTTAGAAC
GGACAGAAAGGATGCGCCTTATAAATGTCCTGTGCAGTGATGAAGCGACCTCAAAACGCT
TCATGATCTAACCGACTCACCTTGCCGTTCCCTCCGCGCCTTAAAACCGGCCGGTCTTGC
GAAAAGCGGGAAACGAGTTTACCCACGGATAGCAGGGAATGTTGCGGCTGGCTAGGGAGC
ATGAAGGTAGATACTCCACGGCTTACCTTTCCGGGGCTCAACATCTAGCCACAGACCTTT
TCGTTAAGCCCACCCCCACTGGATACTGAATCATCAGGGAACCGGACCCAACCAGTTTGG
GCTCGTCCAAGCTTCGGTCTCGTCCCTAAGTGCAAAGATATGGAAAGAGCAGCATAGGTA
TATGGATTATTCTTTTACCACTCGTTTCTTACCGTAACTTACGCAATGGATCACGTGCCG
AGGCGGCGGTACAGCTGTTCGAAGGGCTCTGTCGGGAACGCTAACATCCAGCCGGTAAAT
TCCAAACTAGGGAAAGGACACGCACTGAATTGAATATAGTCGTGAAGGGTGGTGTAAGTC
GTGCACAGCCCGCATTAAGTACTAAACAGCGTCCAATCTTGATCTACTTACGGCCTGATG
TTCTTCAGCACCTCCTAGCACTGGAGTACTTCGCTATCAATGAGATTAGCACTTTGTACA
TGTCATCCAGCCCGAGTCTGGGGTCCGACAATGCGGTCGCCGATTGGTATCTGCATGTAG
TATTAAACGGAGCTGCCGCGGCTGCGGATTATAGTTCATGTCTTGACGGTCCTCGT
```

```
>DNA-2
ACGAGTCAGGCTACAGCATCTTGACACCAGAATATTATGGATTGGACGCTTCCCACTAAA
TGGAAGACTGTTCGGTCATAAACACTACTAGGAATTCCTCTCCAGTCATCATGTTCGATC
GTCTAGCAGCAATCTCTTCCGATCGATATTTGCGCGTGACTCAGGCGAGCCCATGACAGC
TTCTCCCCGTGAGAACCACGACTAGAAGTTATCTGTTGAGCTGCTAGCTTCGTGGCCCGG
CCATGGTAGTAGCGGCTCACTCGCGCTAACTTTGCCTGCTCGAGAAAACGGGCGAAACAC
CCAGCAACACAAGCCACTTAATTTGTTGATAGATAATAAGATCAGGTTATTAGTCGCTCT
GCACTTACTTTAAGTGCCAACTATGCTGTATCGGCCAGGGTGAAAACGGGTGCCGCCACT
TCAGTGTGTCGGAGTCTGCTGACGGATTAGGGCACAGACGTATGGTTATATCCTAAGGTA
GTGTGTCAATGTACTGGGGACAAAGTCAGTGGGCACCGCATCAGGAGTGCAACCTCCGCT
AGTACCGACTCGTCAATGCTTTGAGCGATGGCTTGCGCTCCCAAATCCTTAAGCTTTTAT
GCATTCGGCTCTGGCCCTCAGGCCTGACCTGGAATTTCATCGGAAACGCCTTAACCGACA
TTACATCGACACCAAGATCCCGACGCTTCATGCGGAGACGATAGAGACTCTAACCAAGAA
TAAAAGGAGTAGTCCCTAATCTACTGAAACGGGGATACCTCAAATCACGGGAATGCGTTA
CTGACCCGCTATGTGAGGCTCGGATCACCCTCGTTCTATTGCCTTGTAATCATGGTGGGG
CGGCGGAGCGGGATTAGAGGGTGTCCCTAATGTGAGTAGATCTGTAGTAATGATACGTCT
CCTCAATATGAGGCGTATTGCAGGTCACAGCACAGGGAGATTTCGGCGCACCCAGCCGAG
TTGCCTCCGTCGTTGTTTAGGTATATGCATAACTGCTCACGACAAATACAGCAGAGCCTA
CGTTGGGTTATCGAATCCTTGTGGACAAGAAGCTTCTTCATGTCTTGACGGTCCTCGT
```

>DNA-3

ACGAGCAGCCAGTACCGTCTGGTATTTCCATCAAGTTAGCTTGACACCCAATCAATCTAT
GACGCCCCAGAGCTTGCGATAACTTTGATGCGGTCCTGTACTCGATGACCCTGCGTATCT
AGAACGCGTGTGATAGACGTGGCCAATAACAGGAGGAACTATTCCTTGGTAGCCAATGGC
ACTAGTGTCGGATGTACTTGATCAACCAATACGTCAAGCACAGACCTTCCTTCTCCGGAA
GGTTAGCTATAACAAAGAAGATCATTCGGACGGGGCCGCTAAATGTCCCTGGAGTTTGAG
GATATGAATCTTCACACCACGTGTCATCATCTTGGCGCGTGTAGCAACGAGAAAACACAC
TCTAATGGATCCAGCCGACCTCATACGAGTTAAGTGCTCAATTAGGGTGAAAGTAGGAAC
TGCATCAAGGCTTCCGAGCCCGTCCCCGGTTCCCTGTGCGAGATGCCGCTGGGGTGCCTG
ACCGTTGTTTCGTATTGGGTGTTAGACCGTATCCACGGTGTTTCTAATACTGGCAAGCGC
CGAGGCTATAGCAAGTCCGCTCGT

>DNA-4

ACGAGCAGCCAGTACCGTCGACACGCTTTCGTCGTAAATACTAGCTAGGCTGTACTGCTT
TCACATTGTCCACGGGCACAGGACCGGGGCGGACTTCCCAGCTCACCTCTTGGTGAAACC
GGGAACTCGAATGATCTTTCTTCTAATCATGATTAGGGCAGTCGTCGAAGGAGCCGTTAG
ATTGTGGGCGGAGATTGCAGAAACCACGCGTCAACTAAGAAGAGTCACTGGACGATGGAG
TAGCGCACCTATCCGATACTCAGAATCTCTGGAGGAACGTATGGCATTTGTCCCGATAGG
ATCTTCTCGTTACGTGTGAGTCTTACATCGCGAAGCATACTATGAATACATTAGGTACCC
TGATCGACCTCGGGATTAACGCCGTAATTACTGAGGTTCACTGTTATCAAAACCGCCAGC
TCCGCCGCTTATGTGTTTTATAAATCCCACGCAGGTAGTAGCACTAGCAGTCTCGAAAGC
CCACTACATCGATATGGCCTTTAAGAGCGATGCAGATTGTTGCGCGCCGGCGGCTATAGC
AAGTCCGCTCGT

Data S2. Oligonucleotide sequences

>DNA12ba (reverse (cDNA) primer for DNA-1 and DNA-2)
ACAGTTCACGAGGACCGTCAAGACATG
>DNA12fo (forward primer for DNA-1 and DNA-2)
AGAGTACACGAGTCAGGCTACAGCATC
>DNA34ba (reverse (cDNA primer for DNA-3 and DNA-4)
ATTCGTCACGAGCGGACTTGCTATAGCC
>DNA34fo (forward primer for DNA-3 and DNA-4)
CAAATTCACGAGCAGCCAGTACCGTC
